# Geography, assortative mating, and the effects of sexual selection on speciation with gene flow

**DOI:** 10.1111/eva.12296

**Published:** 2015-08-26

**Authors:** Maria R. Servedio

**Affiliations:** ^1^ Department of BiologyUniversity of North CarolinaChapel HillNCUSA

**Keywords:** allopatry, assortative mating, migration, secondary contact, sympatry, two‐island model

## Abstract

Theoretical and empirical research on the evolution of reproductive isolation have both indicated that the effects of sexual selection on speciation with gene flow are quite complex. As part of this special issue on the contributions of women to basic and applied evolutionary biology, I discuss my work on this question in the context of a broader assessment of the patterns of sexual selection that lead to, versus inhibit, the speciation process, as derived from theoretical research. In particular, I focus on how two factors, the geographic context of speciation and the mechanism leading to assortative mating, interact to alter the effect that sexual selection through mate choice has on speciation. I concentrate on two geographic contexts: sympatry and secondary contact between two geographically separated populations that are exchanging migrants and two mechanisms of assortative mating: phenotype matching and separate preferences and traits. I show that both of these factors must be considered for the effects of sexual selection on speciation to be inferred.

## Introduction

The fascination that biologists have long had with speciation has increased in recent decades as we have gained a better and better understanding of the complexity of this engine of biodiversity. One of the most intriguing aspects of speciation is how it might proceed under the most difficult of circumstances, namely in the face of gene flow. Gene flow poses an especially interesting problem for evolutionary theorists because it counters the forces driving differentiation between species, leading to continued cohesion across them. The problem of speciation with gene flow also has conservation implications; the onset of gene flow after a period of allopatric divergence can erode differences between species or races, posing important questions regarding how species differences can be maintained, or how speciation may continue to proceed.

Speciation in the presence of gene flow, in sexual species, can be driven by two general processes: (i) nonrandom mating that results in assortative mating, the mating of like with like, which reduces gene flow between groups, and (ii) divergent selection, which favors extremes in a population and thus can lead to a splitting of the species (e.g., Kirkpatrick and Ravigné 2002; Coyne and Orr 2004; Gavrilets 2004; selection against hybrids can fall into this category, e.g., Dobzhansky 1937; Muller 1942). To understand when speciation may occur in the face of gene flow, one must understand the joint action of both of these processes.

Assortative mating and divergent selection are, however, not necessarily completely independent components of the speciation process. The mechanisms that generate assortative mating, in almost all cases (except, e.g., symmetric grouping models, Norvaišas and Kisdi 2012), generate differential mating success among genotypes, that is, sexual selection. To understand the importance of this, first it is critical to keep in mind that sexual selection can be a potent evolutionary force, in fact one that is often more powerful than viability selection (e.g., Hoekstra et al. 2001; Kingsolver et al. 2001). Just as sexual selection may drive the evolution of traits within a population, it has the potential to lead to differences between species and has often been speculated to be an important factor in the speciation process, especially in allopatry (West‐Eberhard 1983; Panhuis et al. 2001; Ritchie 2007). The role of sexual selection in speciation with gene flow, however, is less clear; some studies suggest it can drive the speciation process, while others suggest it can inhibit it, under different conditions (e.g., Bolnick and Fitzpatrick 2007; Mallet 2008; Nosil 2008; Maan and Seehausen 2011; Smadja and Butlin 2011; Servedio and Kopp 2012).

In this paper, I maintain that to understand the role that sexual selection plays in speciation with gene flow, it is critical to consider two key factors. First, the geographic context in which gene flow occurs can be very important, as it can alter the patterns of selection that lead to divergence. Second, there are two fundamentally different mechanisms by which assortative mating can occur, even when only considering those forms of assortative mating that act through mate choice (as I do here): phenotype matching and separate preferences and traits. These two mechanisms generate sexual selection in different ways. As part of this special issue on the contributions of women to basic and applied evolutionary biology, I will review theoretical research addressing how the geography of speciation and type of assortative mating interact to affect the role of sexual selection in speciation with gene flow, with an emphasis on synthesizing my own views and contributions to our understanding of this problem, particularly in the case of secondary contact.

## Geography and speciation with gene flow

The geographic background upon which the process of speciation proceeds has long been considered the primary determinant of the ease of speciation, but in recent years, there has been a growing recognition that geographic classifications capture only part of the picture (Butlin et al. 2008; Fitzpatrick et al. 2009; Marie Curie Speciation Network 2012). In a simplistic view, the key distinction between the geographic categories of allopatric, parapatric, and sympatric speciation can be thought of as whether gene flow between incipient species is possible; allopatric speciation occurs in the absence of gene flow, while parapatric and sympatric speciation assume the occurrence of varying degrees of gene flow (Mayr 1963). We now understand that there are finer geographic distinctions that can have important effects on the likelihood of speciation (see Coyne and Orr 2004; Fitzpatrick et al. 2009). Furthermore, even within cases of speciation with gene flow, the geographic context can affect patterns of gene flow, which can ultimately have important effects on the likelihood of divergence (Servedio and Kirkpatrick 1997; Gavrilets 2004; Kisdi and Prikopil 2011; Rettelbach et al. 2013).

**Figure 1:**
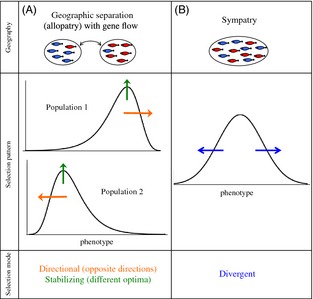
Schematic of the modes of selection necessary to drive speciation with gene flow in the scenarios of (A) allopatric populations that are exchanging migrants and (B) sympatric speciation. The pictures associated with the labels for ‘geography’ show discrete traits for the purpose of illustration, but these scenarios are also relevant for quantitative traits, as shown under ‘selection pattern’. The populations in (A) are drawn as if they have already diverged to some extent. The arrows on the figure are color coded to match the descriptions of selection under ‘selection mode’.

As mentioned in the Introduction, one of the essential components of speciation with gene flow is divergent selection. Selection must be divergent, that is, between the incipient species. If the diverging pair of populations is, however, geographically separated but undergoing gene flow through the exchange of migrants (Fig. 1A, top, note that I still refer to these as ‘allopatric’ as a shorthand), the selection regime within each of the diverging populations can potentially lead to speciation if it is directional, in opposite directions in each population (Fig. 1A, middle, orange arrows). In other words, this opposing directional selection within each population approximates divergent selection when both populations are considered together. Similarly, selection within each population could potentially be stabilizing, but for different optima (Fig. 1A, middle, green arrows). Several theoretical studies have considered ecological speciation between two populations exchanging migrants using ‘two‐island’ models with these types of selection regimes (often including intrinsic selection against hybrids as an additional force favoring divergence, e.g., Felsenstein 1981; Liou and Price 1994; Servedio and Kirkpatrick 1997; Proulx and Servedio 2009). On the other hand, sympatric speciation, which occurs within a single population, requires that selection be divergent *per se* (Fig. 1B, middle). This selection pattern can emerge either as a result of disruptive selection resulting from, for example, bimodally distributed resources, or even because of competition among individuals when resources are unimodal (e.g. Dieckmann and Doebeli 1999; Bürger et al. 2006; Pennings et al. 2008).

The differences in the mode of selection required with and without geographic separation are important, because these modes may differ in how common or how strong they are in nature. Kingsolver et al. (2001), for example, found that, although more data are needed to measure it accurately, current evidence suggests that stabilizing selection and disruptive selection may be weak, while directional selection is often fairly strong in natural populations (see also Kingsolver and Diamond 2011). More speculatively, one could imagine that a vicariance event within a landscape or colonization of a new area may not infrequently lead to the patterns of selection illustrated in Fig. 1A (directional in different directions, or stabilizing for different optima), due to the presence of environmental gradients or divergent environmental conditions.

Sexual selection, like natural selection, can take on different modes during the process of assortative mating. Sexual selection is not static, but changes in a population as the relative proportions of genotypes in the choosing sex changes; that is, sexual selection is inherently frequency dependent. Because of this, directional, stabilizing, or disruptive sexual selection can arise during speciation, depending on the mechanism of assortative mating and the distribution of phenotypes already present in the population, the latter of which can be strongly affected by the geographic context of the speciation event. As I discuss in the following sections, in some cases, the mode that sexual selection takes can drive divergence between populations, but, in others, it can be a powerful force preventing or inhibiting speciation or the maintenance of species differences.

## Modes of sexual selection and the mechanism of assortative mating

Theoretical evolutionary biologists have primarily considered two mechanisms of assortative mating by mate choice: phenotype matching and separate genetic determination of preferences and mating traits (hereafter simply called ‘traits’). (Note that assortative mating can also occur as a byproduct of habitat choice or temporal isolation, which can also generate sexual selection but which I do not concentrate on in this review). During phenotype matching, the choosing sex (which I will call females) prefers to mate with males that match its own trait phenotype; this trait could be a display, such as coloration, a morphological character such as body size, or even a trait that constrains mating such as chirality in snails (e.g., Johnson 1982). When there are instead separate preference and trait loci, the traits might again be functional (e.g., body size, tail length), but if they are ornamental, they may be expressed more commonly in only one sex (e.g., the male). The preference/trait mechanism may therefore comprise a wider variety of mating displays, including potentially across a greater range of sensory modalities, than phenotype matching might typically include.

The mechanisms of phenotype matching and separate preferences and traits generate frequency‐dependent sexual selection in different ways. Because preferences during phenotype matching are determined by the trait itself, phenotype matching generates positive frequency‐dependent selection based on the frequency of the trait phenotype. If traits are normally distributed, for example, the positive frequency‐dependent selection generated by phenotype matching will be stabilizing around the trait mean (see models of this process discussed below). If instead the distribution of traits follows the pattern in Fig. 1A (skewed in different directions in different populations), this positive frequency dependence can produce a selection pattern that is effectively divergent across the two populations.

In contrast, when preferences and traits are determined by separate loci, sexual selection becomes positive frequency‐dependent based upon the frequency of the preference, rather than the frequency of the trait. Preferences that are centered around the trait mean can ultimately generate either stabilizing or disruptive selection, depending on the variance in preferences versus traits (reviewed in van Doorn et al. 2004; Weissing et al. 2011). Specifically, if such preferences have a relatively low variance relative to the trait, they will generate stabilizing selection on trait phenotypes. If, however, preference variation is large enough, competition among individuals with different traits may cause disruptive selection on trait phenotypes, in a way analogous to the disruptive selection generated on traits due to competition over resources under wide resource distributions (van Doorn and Weissing 2001; see Fig. 3 in Weissing et al. 2011). On the other hand, when preferences are displaced from the trait distribution, sexual selection can be directional. To obtain the pattern of disruptive selection, by sexual selection alone, shown in Fig. 1A (orange arrows), preferences would have to be displaced beyond the trait means in different directions in each diverging population.

Because distributions of traits and preferences may change depending upon the geographic and historical context of speciation, as well as through time, the mechanisms of phenotype matching and separate preferences and traits can generate different modes of sexual selection at different points in the speciation process. I briefly review some of these findings for the case of sympatry and then go in more depth into the case of two allopatric populations linked by migration during secondary contact. Note that, there are other common geographic contexts, including parapatry, secondary contact with distinct hybrid zones, and secondary contact with continent‐island patterns of migration, that are not covered here but in which sexual selection may also be generating interesting forces both favoring and opposing divergence (e.g., Kirkpatrick and Servedio 1999; Kirkpatrick 2001; M'Gonigle and FitzJohn 2010; Rettelbach et al. 2013). These are important contexts and worthy of equal consideration in a broader review.

## Sympatric speciation and secondary sympatry

The scenario of sympatric speciation starts with a single population, which one can imagine might often have unimodally distributed traits and preferences. Under phenotype matching, a unimodal distribution of traits would, as discussed above, generate stabilizing sexual selection. Sympatric speciation, however, requires exactly the opposite type of selection, disruptive selection, to proceed (Fig. 1B). The stabilizing sexual selection generated by phenotype matching can, by opposing any disruptive ecological selection, either prevent sympatric speciation completely or cause the assortative mating that evolves during the process of sympatric speciation to stall at an intermediate level (e.g., Matessi et al. 2001; Gourbiere 2004; Kirkpatrick and Nuismer 2004; Otto et al. 2008; Pennings et al. 2008; Sadedin et al. 2009). If trait frequencies are sufficiently skewed within the initial population, the positive frequency‐dependent selection generated by phenotype matching can also have a substantial directional component, leading to the loss of trait variation and thus preventing the evolution of assortment (e.g., Gourbiere 2004; Schneider 2005; Bürger and Schneider 2006; Bürger et al. 2006; Schneider and Bürger 2006; Otto et al. 2008; Pennings et al. 2008). The sexual selection generated during phenotype matching thus may generally inhibit sympatric speciation in its earliest stages.

If the distribution of trait phenotypes in sympatry was bimodal, however, the positive frequency‐dependent sexual selection generated by phenotype matching could instead be divergent. Bimodal trait phenotypes could arise either if sympatric speciation were to proceed under phenotype matching despite the initial stabilizing (or directional) selection that it generates, or if populations divergent in their trait means were to come into secondary sympatry. The model of the evolution of assortative mating during sympatry by Otto et al. (2008), for example, nicely demonstrates this effect using a diploid trait controlled by a single locus. They show that when heterozygotes are common they will be favored, on the whole, by the sexual selection generated by phenotype matching; this can tend to restrict the evolution of a modifier that strengthens assortative mating. When, however, populations have diverged in allopatry and come into secondary sympatry, heterozygotes may initially be rare, especially if there is already any assortative mating. In this case, Otto et al. (2008) show that phenotype matching will cause sexual selection to further favor both homozygotes over the heterozygotes, promoting the evolution of even stronger assortative mating. This has the potential to lead to the evolution of complete assortment (see top right of fig. 4 in Otto et al. 2008; see also Doebeli 1996; Matessi et al. 2001; Arnegard and Kondrashov 2004; Pennings et al. 2008).

Sympatric speciation with separate preferences and traits is likely to be much more difficult than with phenotype matching, primarily because the large amount of preference variation necessary to generate disruptive selection on traits may be very difficult to achieve (reviewed in Weissing et al. 2011). In the scenario of sympatric speciation, one way to generate sufficient preference variation would be if preferences themselves were under disruptive selection (see van Doorn et al. 2004), but this may be rare in natural populations.

## Allopatric populations with migration—phenotype matching

If populations that have undergone trait divergence in allopatry begin to exchange migrants, one would expect trait distributions could be skewed in opposite directions, or in the case of a discrete trait, different trait alleles may initially be at a high frequency in each divergent population. In this case, the positive frequency‐dependent sexual selection generated by phenotype matching will tend to have a directional component, in opposite directions, in each population, matching the pattern of selection that will maintain, or further promote, divergence between populations as shown in Fig. 1A (orange arrows; there can also be a stabilizing component for different trait means, green arrows). In Servedio (2011), I used a population genetic model of two populations undergoing secondary contact by migration to explore the ability of phenotype matching alone to generate enough sexual selection to both allow trait divergence and to promote the evolution of yet stronger assortative mating. To isolate the evolutionary effects of assortative mating by phenotype matching, viability and fecundity selection were assumed to be absent; there was no local adaptation of traits or search costs associated with mate choice. To guarantee that the results were not due to the low fitness of hybrids (reinforcement), haploids were used so that no intermediate phenotypes were generated. Females were simply assumed to have a preference for matching the trait allele (T_1_ or T_2_) that they also carried.

**Figure 2:**
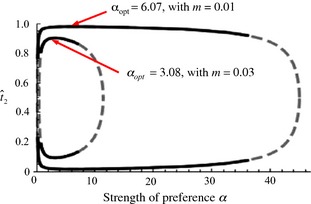
The amount of trait divergence between two populations exchanging migrants under assortative mating by phenotype matching, as a function of the strength of preferences, α, for two different migration rates (*m *=* *0.01 and *m *=* *0.03). The lines shown are equilibrium frequencies of the trait T_2_ in population 2; as the identity of the ‘local’ trait is interchangeable in a model with only phenotype matching, if T_2_ is at the frequency shown by the top line in population 2, then T_1_ will be at the same frequency in population 1. The difference between the two lines can thus be considered a measure of the divergence between the traits across the two populations. The solid black line is a stable equilibrium line reached from the assumption of secondary contact between divergent populations and the dashed gray line is an unstable equilibrium. Stable lines of equilibrium where both populations are fixed for T_2_ or for T_1_ and an unstable equilibrium at 0.5 are not shown on the figure. The value of α that leads to the maximum amount of divergence between the populations, α_opt_, is labeled for each migration rate (differentiation cannot be maintained if migration rates are too high). Redrawn with permission from Servedio (2011).

Despite the simplicity of this model, it yields some unexpected results; phenotype matching alone can maintain trait divergence, but this divergence starts to decline if preferences are too strong (above the points labeled α_opt_ in Fig. 2), and as preferences continue to increase in strength it eventually collapses altogether. Figure 2 shows the frequency of the T_2_ allele at equilibrium, as the strength of the preference, α, that females have for their matched trait, varies on the *x*‐axis (α determines how much more likely a female is to mate with a male that matches her trait if she were to encounter males at equal frequencies; an α of 0 would correspond to random mating and a high α would correspond to very strong preferences). If the strength of preference does not vary between populations, the frequencies of the traits evolve to be symmetric between populations when divergence is maintained; the difference between the black curves in Fig. 2 thus represents the amount of trait divergence that the populations experience (see figure legend). As can be seen in Fig. 2, with very weak preferences, and hence mating that is close to random, trait divergence cannot be maintained, but as preference strength increases, traits initially become more divergent. Interestingly, however, a yet‐further increase in preference strength causes the amount of divergence to drop (once α_opt_ is crossed); when preferences are very strong, no trait differentiation can be maintained.

The decline in trait divergence with strong preferences can be explained by the fact that positive frequency‐dependent sexual selection, which is the only evolutionary force leading to divergence in this model, starts to decline as preferences become too strong. This can be understood by considering the mating success of rare males. When preferences are absent, rare males mate in proportion to their frequencies, so there is no positive frequency‐dependent sexual selection. Importantly, this is also true when preferences are very strong; in this case, rare females have such a strong preference for rare males (just as common females do for common males) that they will not accept mates that do not match their phenotype. It is only when preferences are of an intermediate strength that they generate positive frequency‐dependent sexual selection, so it is only at these intermediate strengths that sexual selection can oppose the homogenizing effects of migration to lead to divergence.

Interestingly, the value of the preference strength that maximizes the divergence between traits across the populations, α_opt_, is also the value that will evolve if assortative mating strength is allowed to vary evolutionarily (Servedio 2011). This occurs because an allele that causes more trait divergence will naturally become genetically associated with the more common allele in each population and thus will increase in frequency through indirect selection as the trait undergoes divergent sexual selection (for a full explanation see Servedio 2011). Because preferences will evolve to the strength that maximizes divergence, assortative mating can indeed evolve due to the presence of phenotype matching alone; it can evolve from a very low preference strength to this optimum. However, it will also tend to evolve from a very high preference strength, if it were to reach a high strength by some other means (e.g., perhaps in allopatry), down to this optimum. Populations in this situation will thus tend to reach an evolutionarily stable intermediate level of assortative mating (e.g., α_opt_ ≈ 6 or 3 in Fig. 2).

These properties of phenotype matching can also be seen in models that contain more biological complications. If traits are locally adapted, for example, they will not necessarily lose variation if preferences are too weak or too strong (they will be in migration/selection balance), but divergence will still be maximized at the intermediate preference strength that leads to the strongest positive frequency‐dependent sexual selection (Servedio 2011; preference strength is still seen to evolve to the value that maximizes divergence, α_opt_, in this case). Proulx and Servedio (2009) examined the evolution of 12 different combinations of preferences for traits that were locally adapted, influenced hybrid fitness, or matched the female's trait value, using a two‐island model. They similarly found that when preferences were very strong, there was a decrease in the rate of spread of any mating strategy that included phenotype matching of a locally adapted trait, although they did not examine the evolution of the strength of assortative mating.

Assortative mating by phenotype matching may likewise generate the most divergent sexual selection across populations at an intermediate preference strength during the process of reinforcement. Reinforcement, defined as the evolution of premating isolation as an evolutionary response to selection against hybrids or hybridization (Dobzhansky 1940; Servedio and Noor 2003), has garnered much attention as a process whereby species boundaries can be bolstered after secondary contact. Reinforcement models are considerably more complicated than the basic model of Servedio (2011), partially because of the increased number of genotypes that can both act as cues for mating (while in males) and can express mating preferences (while in females). A variant of a reinforcement model, in which phenotype matching occurred across two loci (N and M), was also examined in additional analyses in Servedio (2011); here, ‘purebred’ N_1_M_1_ and N_2_M_2_ females preferred matching males, while hybrid N_1_M_2_ and N_2_M_1_ females had no preference. Intermediate preference strengths were found to lead to the most divergence at the M and N loci both when hybrids had low fitness simply due to lower mating success, and when viability selection against hybrids was explicitly introduced.

Because the reduction in divergent sexual selection across populations with strong phenotype matching results from the very basic phenomenon of the increased mating success of rare males, it should be general to many models of phenotype matching with spatial structure (although there may be situations where the effect is masked, including by activity at other loci). Indeed, I have now recognized this phenomenon to be present in some older models of my own (e.g., the models of phenotype matching of a locally adapted trait with male and female preferences in Servedio 2007) and have documented it in phenotype matching reinforcement models with diverse conditions such as a learned trait (Olofsson et al. 2011) and with within‐generational learning about prospective mates (Servedio and Dukas 2013). Whether it is a notable phenomenon in natural systems that use phenotype matching will depend in part on whether preference strengths in nature are strong enough to be above the preference strength α_opt_ that leads to the peak in trait divergence. With migration rates in the low single digits (e.g., 1–3%), the preference strengths at which trait divergence would start to decline, while high, would be potentially measurable (e.g., females being 3–6 times more likely to mate with preferred males in a choice trial). Of course, in cases where preferences may have indeed been strong enough to eliminate population differentiation there would be no divergent populations to study; this does not mean the phenomenon is not biologically relevant, but just that it will not be observed. In populations with low density, it is also possible that females may alter their behavior to be less discriminatory, even if they have strong underlying preferences, when rare males are too rare. This may retard the reduction in trait divergence with very strong preferences, but whether it would eliminate it would depend on how rare males must be before loss of discrimination would occur, as well as on the degree of this loss of discrimination.

The relevance of these effects will also, of course, depend on how often phenotype matching occurs in natural populations. While this question is underexplored, there is at present a dearth of strong evidence to suggest that it is common (M. Kopp, M. R. Servedio, T. C. Mendelson, R. J. Safran, R. L. Rodriguez, E. S. C. Scordato, L. B. Symes, C. N. Balakrishnan, M. E. Hauber, D. M. Zonana and G. S. van Doorn, unpublished manuscript). It turns out, however, that sexual imprinting, provided that it is on kin, can be a close proxy for phenotype matching and is found across many taxa (reviewed in Verzijden et al. 2012). Females that establish a mating preference by sexually imprinting on a maternal or paternal trait will very often be matching their preference to the trait alleles that they themselves carry (even if these traits are not expressed). These forms of imprinting have the potential to behave very similarly to phenotype matching in both sympatric speciation (Verzijden et al. 2005) and reinforcement (Servedio et al. 2009) models in certain cases. Maternal and paternal imprinting do, however, differ in several important properties, including in the fact that, under polygyny, paternal imprinting enhances the effects of sexual selection by leading to exaggerated preferences (Tramm and Servedio 2008); this can lead to interesting differences in the specifics of their effects on trait divergence (Verzijden et al. 2005; Chaffee et al. 2013; Yeh and Servedio 2015).

## Allopatric populations with migration—separate preferences and traits

Ever since the earliest models of the process, Fisherian sexual selection has been speculated to lead to species differences between allopatric populations (e.g., Lande 1981; West‐Eberhard 1983; Panhuis et al. 2001; Ritchie 2007; Uyeda et al. 2009). Lande (1981) and Kirkpatrick (1982) demonstrated using quantitative genetic and population genetic models, respectively, that female mating preferences and male traits would become exaggerated during sexual selection due to the presence of the statistical correlation (linkage disequilibrium) that naturally arises between them. Sexual selection increases the trait value or frequency, which leads to indirect selection increasing the preference value or frequency via this linkage disequilibrium, which in turn leads to more sexual selection on the trait, and on and on in a feedback loop (see Fisher 1930). Lande (1981) and Kirkpatrick (1982) both find that this process, coupled with viability selection on the male trait, results in lines of equilibria representing different combinations of preference and trait values or frequencies (although these lines occur for different reasons, Barton and Turelli 1991; note that, the lines can reduce to a point if preferences are costly, e.g., Pomiankowski 1987). When there is strict allopatry, different populations can end up on different places on the line of equilibria and hence have divergent traits and preferences (even potentially when there are preference costs, see Uyeda et al. 2009). In cases in which the line of equilibria is unstable, runaway sexual selection could potentially drive populations to widely divergent trait and preference values (a runaway can also occur even with directly selected preferences, Hall et al. 2000). Divergent traits and preferences between populations could result in premating isolation and hence potentially speciation.

Less well appreciated is the issue of whether this process of divergence can occur if there is any migration between allopatric populations, resulting in gene flow. Lande (1982) and Payne and Krakauer (1997) both determined in clinal models that in the absence of local adaptation, Fisherian sexual selection alone could not maintain the differences in preferences and traits. This was confirmed in a two‐island population genetic model by Servedio and Bürger (2014) and is consistent with the difficulties faced by sympatric speciation models that consider preferences and traits (e.g., Weissing et al. 2011), discussed above.

In Servedio and Bürger (2014), we also considered a situation much more favorable to speciation—one which quite a number of speciation researchers that I informally polled (including myself!) thought would lead to sexual selection promoting speciation, despite gene flow. In this model, we assumed that the male trait was, itself, under divergent selection, so that allopatric populations had evolved a preference for a locally adapted male trait before migration began between them. Traits that are both under divergent ecological selection and used as mating cues during assortative mating have been well established to result in the evolution of premating isolation particularly easily (Gavrilets 2004), in comparison with cases in which different sets of loci perform these two different functions. Because of the ease with which they result in speciation, they have been termed ‘magic traits’ (Gavrilets 2004); this name implies that they may be rare, but they are being found in more and more species pairs (Servedio et al. 2011). When preferences are very weak, locally adapted mating cues will be close to migration/selection balance, so divergence between the populations will be maintained. We initially expected that if preferences for the locally adapted trait were stronger in each population (remembering that preferences, like traits, were assumed to have diverged in allopatry), more differentiation in traits would be maintained in each population at equilibrium.

**Figure 3:**
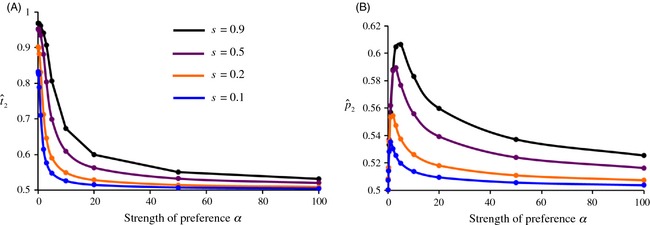
Local trait (*t*_2_) and preference (*p*_2_) frequencies in population 2 at equilibrium, as a function of the strength of preferences, α. Higher values represent more differentiation between the two populations in trait and preference frequencies, while a frequency of 0.5 represents no differentiation. (A) equilibrium trait frequencies. (B) equilibrium preference frequencies [note the range of the *y*‐axis differs from panel (A)]. The migration rate *m *=* *0.01; higher migration results in the same pattern but with each curve at lower values of ${\hat{t}}_{2}$ and ${\hat{p}}_{2}$. The strengths of selection leading to the local adaptation of trait T_2_ are shown in the inset on panel (A), where the locally adapted trait has a relative viability of 1 + *s* in each population. Redrawn with permission from Servedio and Bürger (2014).

We instead obtained the opposite result; stronger preferences resulted in less, not more, differentiation between populations in trait (and sometimes preference) frequencies (Fig. 3, Servedio and Bürger 2014). The explanation of this effect is as follows. First, it is important to keep in mind two facts: (i) with low migration rates or strong local adaptation, the amount of divergence in trait frequencies between populations can be relatively high right after viability selection occurs in the life cycle, and (ii) sexual selection naturally occurs after viability selection (only surviving adults have the opportunity to mate). When there is random mating (no preferences, α = 0 on Fig. 3), sexual selection does not act to change trait frequencies after viability selection, and trait differentiation, as explained above, will reach the value determined by migration/selection balance (recalling that we are interested here in traits that are locally adapted). When instead there are very strong preferences, sexual selection will cause the trait frequencies among successfully mated males to mimic the preference frequencies, as each female will not mate with any male except the type that she prefers. The effects of local adaptation on trait frequencies will therefore be superseded by those of sexual selection.

The key, however, is that preference frequencies in this scenario will always, under Fisherian sexual selection, be more homogenized between populations than trait frequencies will. Both preference and trait alleles will tend to be homogenized between populations by migration. With locally adapted traits, this migration is countered by viability selection to lead to trait divergence. Preference alleles, however, are not under local adaptation. Instead, under Fisherian sexual selection, they evolve only due to the linkage disequilibrium that they have with male traits. Because this indirect selection on preference alleles is mediated by the strength of linkage disequilibrium, it is generally weak (e.g., Kirkpatrick and Barton 1997), and low preference differentiation results. These homogenized preferences will in turn cause sexual selection to tend to be a homogenizing force between populations, and the stronger it is, the more it will counter local adaptation to bring trait frequencies closer together (Fig. 3).

Interestingly, if the strength of sexual selection is itself allowed to evolve in this model, it evolves to random mating, as a result of the fact that alleles for weaker preferences become associated in each population with the locally adapted trait (Servedio and Bürger 2014). This prevents the maintenance, across the populations, of complexes of traits and preferences with high levels of linkage disequilibrium. Thus, strong preferences reduce population differentiation when they are not allowed to evolve, and when they are allowed to evolve, they evolve toward random mating. These combined effects leave very little room for Fisherian sexual selection to contribute toward diversification with this geographic scenario.

By focusing on Fisherian sexual selection, the goal of Servedio and Bürger (2014) was to uncover the effect that would be generated by a process that is present in almost all forms of sexual selection. The fact that preferences place direct selection on traits, and themselves evolve as a correlated response to this selection, is fundamental to any process of sexual selection that includes variable and heritable preferences and traits. It is thus, and I believe rightly, considered a ‘null model’ for sexual selection (Prum 2010). In cases of secondary contact by migration where sexual selection seems to promote divergence, the results of Servedio and Bürger (2014) indicate that a more biologically complex form of sexual selection is occurring. I hope these results will prompt researchers to determine exactly why sexual selection may be promoting divergence in such cases. Note that, while some departures from Fisherian sexual selection can reverse the effect found in Servedio and Bürger (2014), and allow sexual selection to promote divergence, others will still result in sexual selection tending to inhibit trait divergence (see the mixed results of variant models with search costs, viability selection in females, and a best‐of‐n choice strategy in Servedio and Bürger 2014).

One situation of secondary contact in which stronger preferences may have the potential to lead to more trait differentiation between populations is when there is selection against hybrids, that is, when reinforcement is occurring. Kelly and Noor (1996) and Bank et al. (2012), for example, have considered models of reinforcement with very different biological underpinnings, but which assume specific preferences for divergent male phenotypes. In both cases, they discovered that a modifier that effectively increased the strength of the underlying preferences could spread, but that this required hybrids having low fitness. In the case of Bank et al. (2012), modifiers of large effect were even found to be able to lead to complete reproductive isolation. On the other hand, Servedio (2000) used a reinforcement model to consider the evolution of a novel preference for a locally adapted trait characteristic of just one of two diverging populations. When the ancestral allele at this preference locus was for random mating, evolution of the preference would reduce the production of low‐fitness hybrids in the population in which it evolved. In this case, stronger preferences did not necessarily allow reinforcement, measured as an increase in the novel preference, to occur more easily, due in part to selection against the novel preference when it was in the foreign population. In general, the dynamics in models including sexual selection by separate preferences and traits are quite complex, and it is difficult to make general statements about their results; much may depend on the underlying biology of a specific pair of species (e.g., Bank et al. 2012).

## Conclusions

What can we conclude about the effects of sexual selection on speciation in these geographic contexts from these theoretical models? In sympatric speciation, it is very difficult to argue that sexual selection is doing anything but inhibiting divergence in most cases. With phenotype matching, sexual selection is unlikely to contribute to divergence except in the late stages of sympatric speciation or in secondary sympatry, when there is already substantial divergence present (e.g., Doebeli 1996; Matessi et al. 2001; Arnegard and Kondrashov 2004; Gourbiere 2004; Otto et al. 2008; Pennings et al. 2008). With separate preferences and traits, special conditions, such as divergent selection on preferences themselves, must be present to allow enough preference variation for sexual selection to contribute to sympatric divergence (reviewed in Weissing et al. 2011). In sympatric speciation, sexual selection will generally either produce stabilizing selection on traits or eliminate trait variation with both mechanisms of assortment.

In the case of migration between two geographically isolated populations, as in secondary sympatry, sexual selection can promote divergence when assortative mating occurs by phenotype matching; here, matching produces positive frequency‐dependent sexual selection that can be effectively directional in opposite directions in each population, driving trait means apart. As discussed above, however, this selection can be relatively ineffective, because it can drop or even disappear at high preference strengths (Servedio 2011). When assortative mating instead occurs by separate preferences and traits, the most basic form of sexual selection (Fisherian) will fail to maintain trait divergence and will indeed tend to evolve toward random mating (Servedio and Bürger 2014). While certain forms of sexual selection may be able to promote differentiation in this case, sexual selection cannot be said to generally have the property of driving speciation. Over all of these cases and geographies, the role of sexual selection in speciation is not generally positive.

The case of secondary contact between species that have diverged somewhat in allopatry may be of particular interest. First, it may occur relatively frequently, as evidenced by the ability of many species to hybridize when they are in contact (Mallet 2005; Nosil et al. 2009). Second, it has conservation implications if rates of contact are increasing due to anthropogenic change. There are, however, many missing pieces that would need to be gathered empirically to truly address the role of sexual selection in this context, to complement the theoretical work in this area.

First, it is important to have a better assessment of how often there are genetically distinct preferences and traits versus phenotype matching (or a proxy of matching such as sexual imprinting). While the biology of many systems, such as those that are sexually dimorphic, seems to make it much more likely that they have separate preferences and traits rather than use phenotype matching, truly proving that one or the other of these mechanisms exists is not necessarily trivial (reviewed in Kopp et al., unpublished manuscript, see Verzijden et al. 2012 for a review of sexual imprinting). More than one system which has originally been considered to have separate preferences and traits, for example, have been shown by further study to have preferences altered by exposure to the phenotype of the parents, leading to a matching‐like mechanism (e.g., Verzijden and ten Cate 2007; Verzijden et al. 2008; Kozak et al. 2011).

Box 1 **Personal Reflections**

My strategy as a woman in science, early in my career, was to plow on as though being a woman was irrelevant, as ideally it should be. I am not in any way suggesting that I am currently blissfully ignorant of challenges such as glass ceilings and implicit (or explicit) bias, although early on perhaps I was. I was very fortunate to benefit from excellent mentorship at all points in my career. As an undergraduate at Harvard I received a lot of encouragement from Bruce Waldman, A.W. Crompton, and Jeff McKinnon, whom I worked for when he was a graduate student. As a graduate student myself, at the University of Texas at Austin, I worked with Mark Kirkpatrick and benefitted immensely from the high standard to which he held me. My post doc mentors, Alex Kondrashov (at Cornell), Michael Turelli and Sergey Nuzhdin (at UC Davis), and Russ Lande (at UCSD) were also extremely supportive, put a tremendous amount of time into mentoring me and generally aided my transition to an independent career. The only female role model that I had as a mentor was Susan Kalisz (Univ. of Pittsburgh), in whose laboratory I spent 2 years when I was a graduate student. I could not have asked for a more amazing example of a brilliant woman scientist with an excellent work/life balance. If I ever had any doubts that I could have what I wanted in both of these arenas, they vanished in those years.

Obtaining a good work/life balance, for me, faced its greatest challenge when I had children— meaning that as I was not willing to spend less time with my children, it was difficult to figure out how to accomplish as much as I wanted to. Children are a wonderful part of life for women who choose to have them, but keep the struggle to maintain full involvement in a career quite interesting. My children, for example, seem to have figured out that the best way to keep me home on the eve of a foreign flight is to make sure that one of them has a fever of at least 104°, is in the hospital, or is otherwise under medical supervision (they have literally done all three of these!). Fortunately, they have always managed to bounce back by what would have been the second day of my (now canceled) trip. This only scratches the surface of the juggling act. It is certainly possible to have children and a full career. It would be easier, though, if universities and even granting agencies put more policies in place (there are many possible ones) to support academic parents.

Even once the type of assortative mating mechanism is established, it would be difficult to make theoretical predictions about the role that sexual selection may play for a particular species pair without knowing a lot more information. For the case of separate preferences and traits, for example, we might need to know the strength and direction of selection on preferences, preference and trait distributions, the strength and direction of natural selection on traits, the range of preference strengths found between incipient species, and the strength of correlations between preferences and traits, among other factors. For phenotype matching, at a minimum, we would need to know distributions of traits (including skew), the strength of preferences for matched phenotypes, and the costs of searching for mates (e.g., Schneider and Bürger 2006; Kopp and Hermisson 2008).

In general we, as a field, are far from having all of the data necessary to empirically explore many of the predictions from theoretical studies of speciation. The contribution that these theoretical studies provide is a test of our verbal logic as to how the complicated pieces of the puzzle may fit together to lead to speciation; they clarify these chains of cause and effect (Servedio et al. 2014). Theoretical studies have made it clear that just showing empirically that sexual selection currently leads to some reproductive isolation does not prove that stronger sexual selection would allow more, rather than less, trait divergence, or even more easily facilitate population divergence. It is only by a merging of the functions of theoretical and empirical work that we will fully understand the bigger picture of how often, and why, sexual selection contributes to, versus inhibits, the speciation process.

## Data archiving statement

This article has no associated data to be archived.
